# First Principles Calculation for Photocatalytic Activity of GaAs Monolayer

**DOI:** 10.1038/s41598-020-66575-9

**Published:** 2020-06-12

**Authors:** Yilimiranmu Rouzhahong, Mariyemu Wushuer, Mamatrishat Mamat, Qing Wang, Qian Wang

**Affiliations:** School of Physics and technology, Xinjiang University, 666 Victory Road, Urumqi, 830046 P.R. China

**Keywords:** Energy, Two-dimensional materials

## Abstract

Solar energy hydrogen production is one of the best solutions for energy crisis. Therefore, finding effective photocatalytic materials that are able to split water under the sunlight is a hot topic in the present research fields. In addition, theoretical prediction is a present low-cost important method to search a new kind of materials. Herein, with the aim of seeking efficient photocatalytic material we investigated the photocatalytic activity of GaAs monolayer by the first principles calculation. According to the obtained electronic and optical properties, we primarily predicted the photocatalytic water splitting activity of GaAs monolayer, which the result further confirmed by the calculated reaction free energy. More remarkably, predicted carrier mobility of GaAs monolayer 2838 cm^2^V^−1^s^−1^ is higher than 200 cm^2^V^−1^s^−1^ of MoS_2_. Our finding provides a promising material for the development of renewable energy conversion and a new outlook for better designing of a superior photocatalyst for water splitting.

## Introduction

Global energy crisis is well known argent issue at present^1,2^. Photocatalytic catalysts that can produce hydrogen by splitting water under the sun light have been considered as merited materials to solve the energy crisis by converting solar energy to chemical energy^3,4^. This tendency assigns important missions to the scientists to find efficient photocatalytic catalysts that operating well under the sun light irradiation^5^. Many scientists paid their attention on the two-dimensional (2D) semiconductors since 2D materials fulfil the requirements of energy-conversion devices with suitable width of band gap, wider surface area, lower recombination rate of photo-generated electron-hole pairs, and higher absorbance for visible light^6–10^. To date, a series of efficient photocatalytic monolayers such as h- BN^11^, ZnO^12^, WS_2_^13^, TiO_2_^14^, and black phosphorene (BP)^15^ have been fabricated experimentally, while a series of monolayers like GeTe^16^, CdS^17^, SnP_3_^18^, MnPSe_3_^19^, PbSeO_3_^20^ and BC_2_N^21^ *et al*.^22^ were also theoretically predicted as a will realized efficient photocatalytic catalysts.

There are several strict band gap requirements for ideal photocatalytic water splitting materials^23^: (1) Band gaps should be larger than the free energy of water splitting (1.23 eV), while in order to efficiently harvest solar energy the band gap required to lower than 3.0 eV^20^. (2) The minimum conduction band (CBM) should be greater than the hydrogen reduction potential (H^+^/H_2_, −4.44 eV), and the maximum valence band (VBM) should be smaller than the oxidation potential of oxygen (H_2_O/O_2_, −5.67 eV). (3) To avoid surface recombination for carriers, photo-generated carriers must be transferred rapidly and separate efficiently. These strict requirements have been limiting the utilization possibility of numerous 2D materials as photocatalysts for practical hydrogen generation. Thus, scientists by adding sacrificial reagent or loading of catalysts (co-catalysts) try to improve utility of some 2D materials as photocatalysts. Although the widely used co-catalyst noble metal can able to effectively improve the photocatalytic activity, but the associated cost and toxicity of some of them (especially Cr) prohibiting their practical applications^24^. Therefore, current scientific attempts to explore effective photocatalysts have not sufficiently fulfilled the practical demands, and seeking for an efficient 2D photocatalyst is an important mission and a big challenge^25^.

GaAs is one of the most studied applicable semiconductors with the 1.53 eV band gap. GaAs has been used in solar cells, detectors, light-emitting devices, temperature measurements, and spin-charge converters^26^. Recently, GaAs monolayer was theoretically predicated by several scientific groups, and magnetic and nonlinear optical properties of GaAs have attracted interest in scientific community^27–29^. The reported suitable visible light harvesting band gap^30,31^ of GaAs monolayer, larger carrier mobility than GaAs bulk, larger light absorption surface than the bulk, and unexplored photocatalytic activity inspires us to investigate the photocatalytic activity of GaAs monolayer.

In this work, by using density functional theory (DFT), we systematically discussed the photocatalytic water splitting activity of the GaAs monolayer. Electronic and optical results disclosed that the GaAs monolayer has photocatalytic water splitting activity without adding sacrificial reagent, or co-catalysts. The carrier mobility is calculated by the deformation potential theory. The carrier mobility of GaAs monolayer is highly anisotropic, and its magnitude 2838 cm^2^V^−1^s^−1^ is higher than the values of many other photocatalytic monolayer materials, like MoS_2_, BN, and BC_2_N.

## Calculation Details

In order to determine the effects of structure on properties, the properties calculations of GaAs monolayer were carried out by the CASTEP program with DFT based plane-wave pseudopotential^32^. The structure optimizations, phonon calculation and elastic constants were carried out using generalized gradient approximation (GGA) with Perdew-Burke-Erenzerhof (PBE) function^33^, while hybrid function PBE0^34^ employed to determine electronic structure and optical properties. During all calculations, the kinetic energy cut-off for wave function expansion is set as 800 eV. 7 × 7 × 1 Monkhorst-pack k-points were used for Brillouin zone sampling. The other calculation parameters were set as default values of CASTEP program. For all the calculation, we set monolayer parallel to *a-b* plane and perpendicular to *c* direction, and to avoid interlayer interaction the supercell length of 20 Å as vacuum thickness is adopted.

The carrier mobility can be calculated by the equation^16,21^:

$${\mu }_{2D}=\frac{e{\hbar }^{3}{C}_{2D}}{{K}_{B}T{m}^{\ast }{m}_{a}{({E}_{1})}^{2}}.$$

where, *e* is the charge of electron, *K*_*B*_ and *ħ* are the Boltzmann and the reduced Planck’s constants, respectively. *T* is thermodynamic temperature (300 K), m* (${m}_{M}^{\ast }$ or ${m}_{K}^{\ast }$) and *m*_*a*_ are the transverse and average (${m}_{a}=\sqrt{{m}_{M}^{\ast }{m}_{K}^{\ast }}$) effective masses, respectively. *E*_1_ is the deformation potential which is determined by the shift of VBM for holes (CBM for electrons) caused by the small lattice strain (Fig. S2). In this work, all the quantities mentioned above are calculated by the PBE0 hybrid functional approach.

Changes in Gibbs free energy and activation energy are encouraged to investigate the kinetic properties that directly determine the reaction rates^35–37^. There the Gibbs free energy change (Δ*G*) for the hydrogen reduction and water oxidation reaction was calculated by the method^5,38^ which proposed by Nørskov *et al*., according to that method Δ*G* is computed by the following equation^39^:

$$\Delta G=\Delta E+\Delta {E}_{zpe}-T\Delta S+\Delta {G}_{pH}+\Delta {G}_{U}.$$

where Δ*E* is the reaction energy, Δ*E*_*zpe*_ and Δ*S* are the zero-point energy and entropy difference for the absorbed state and gas phase respectively. Here, Δ*G*_*pH*_ = pH × 0.059 eV, Δ*G*_*U*_ = *−eU*, in which *U* is the electrode potential.

## Results and Discussion

### Structure and stability

**Figure 1:**
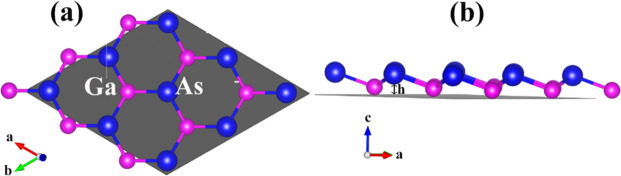
Geometric structure of GaAs monolayer. Top view (**a**) and side view (**b**) of 2 × 2 × 1 GaAs monolayer supercell, where h is the buckling height.

The GaAs monolayer structure is belong to the monolayer honeycomb structure which formed by repeating six membered rings, and one unit cell of GaAs monolayer contains one Ga atom and one As atom, as shown in Fig. 1. Each Ga atom is three-fold coordinated with three As atoms, while the As atom is also three-fold coordinated with three Ga atoms. GaAs monolayer buckling height *h* is 0.41 Å and bond length of Ga-As is 2.41 Å which well matches with other previous work^31^.

Before studying the potential function of the GaAs monolayer, the fabricating possibility and stability of the GaAs monolayer were checked and approved by the following theoretical methods: (1) Cohesive energy of the GaAs monolayer is calculated by *E*_coh_ = (*E*_Ga_ + *E*_As_ − *E*_GaAs_)/2, where *E*_Ga_ and *E*_As_ are the total energy of single Ga and As atoms, respectively, and *E*_GaAs_ is the total energy of GaAs unit cell. The obtained cohesive energy 7.11 eV/atom can be compared to the value of 7.85 eV/atom for graphene, but larger than the values of some typical 2D materials, e.g., silicene with 3.98 eV/atom and black phosphorene (BP) with 3.48 eV/atom^15^. The favourable cohesive energy value demonstrates the fabricating possibility of GaAs monolayer. (2) Dynamical stability is evaluated by phonon dispersion along the high-symmetry points in Brillouin zone, as shown in Fig. S1. The phonon dispersion has no imaginary frequency modes, which reveals the kinetic stability of the GaAs monolayer. (3) The mechanical stability is analysed by calculating elastic constants. The elastic characteristics of hexagonal structure are determined by three independent elastic constants C_11_, C_12_ and C_66_. The obtained results of elastic constants C_11_ = 38.05 N.m^−1^, C_12_ = 9.62 N.m^−1^, and C_66_ = 14.22 N.m^−1^ meet the hexagonal structure mechanical stability criteria^36^: C_11_ > |C_12_|, C_66_ > 0. The result means that GaAs monolayer is stable under mechanical deformation. Therefore, based on the characteristics of the energetically favourability, phonon dispersion with positive phonon modes and elastic constants that meet the stability conditions, GaAs monolayer may be synthesize in experiments.

### Electronic structure properties

**Figure 2:**
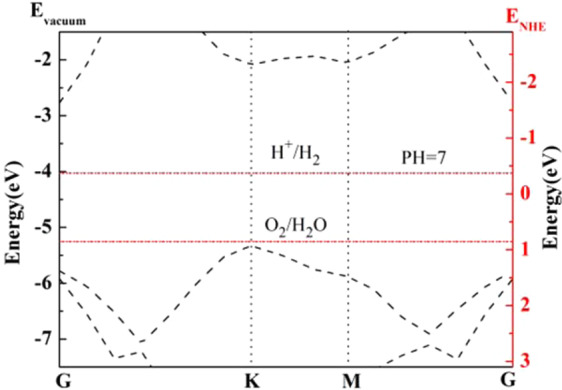
Band structure of the GaAs monolayer.

Since the nonlocal exchange-correlation functional is more accurate to evaluate band gaps of semiconductors and insulators as compared to GGA functionals based on Kohn-Sham schemes, we calculated the band structure by PBE0 as shown in Fig. 2. It’s clear to seen, GaAs monolayer is an indirect band gap semiconductor with 2.56 eV band gap value, and the result is consistent with previous work^31^. From the band structure of the GaAs monolayer, we can clearly see that the VBM is at K point, while the CBM is at G point, respectively.

To assess the satisfaction of band edge requirement for photocatalytic water splitting, band edge positions of GaAs monolayer were further compared with the water splitting redox potentials for hydrogen evolution (−4.03 eV) and oxygen evolution (−5.26 eV). As plotted in Fig. 2, the position of VBM (−5.32 eV) and CBM (−2.76 eV) are straddled the redox potential of water, in neutral environment (pH = 7). From the relationship of redox potential with pH value (−4.44 + pH × 0.059 eV for H^+^/H_2_; −5.67 + pH × 0.059 eV for O_2_/H_2_O) we confirm that in acidic environment (pH < 7) the VBM position states under the oxygen evolution potential. Obviously, the results disable to fullfil the requirements of photocatalytic water splitting. However, the results indicate that the GaAs monolayer possesses splitting activity at neutral environment.

During the photocatalytic water splitting process, the external potential that can drive all reaction process will be provided by photo-generated electron and hole^40^. In this work, we calculated the external potential at neutral environment (pH = 7). For hydrogen reduction reaction, the external potential (*U*_e_) is defined by energy difference between the hydrogen reduction potential (H_+_/H_2_ potential in Fig. 2) and the CBM, and the obtained *U*_e_ value is 1.29 V. While for the water oxidation, the external potential (*U*_h_) is defined by the energy difference between the oxygen evolution potential and the VBM, and the obtained *U*_h_ value is 0.04 V. In contrast to the value of the *U*_h_, the large value of the *U*_e_ enables GaAs monolayer to possess an excellent water splitting property.

### Effects of external strain on band gap and edge positions

**Figure 3:**
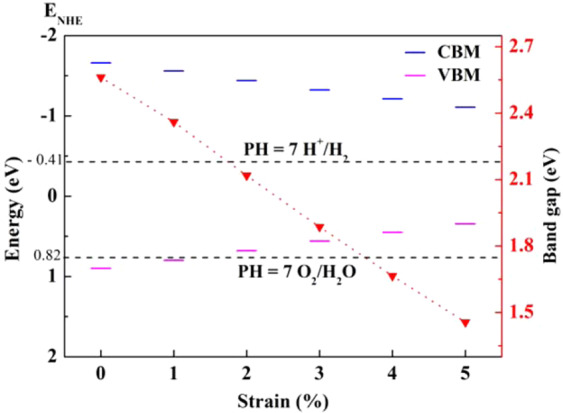
Variation of the GaAs monolayer band gap and band edge position with biaxial strain.

The bang gap and band edges are important parameters for photocatalytic water splitting. Furthermore, except from the reaction pH environment, modulating the bang gap and edge position is also one of the effective methods to improve the effectiveness of photocatalytic water splitting. At present, introducing external strains is a feasible method to modulate the band gap and edge positions^41^. Therefore, we further investigated the effects of external strain on the band gap and edge positions, and the related result is depicted in Fig. 3. Here, the strain (ε) is defined as ε = (l − l_0_)/l_0_, where l and l_0_ are parameters for strained and nonstrained lattices respectively, and the considered maximum strain is 5%.

As displayed in Fig. 3, the external strain has dramatic effects to the band gap and edge positions of GaAs monolayer. When the monolayer subjected to the biaxial tensile strain, the CBM is shifted downward while VBM is shifted upward gradually. When the strain reaches 1%, the band edge position still satisfies the water splitting requirements. However, when it comes to the band gap, band gap narrows down with increasing of the strain. The band gap is reduced to 1.46 eV, when strain reaches maximum of 5%. The results indicate that the application of an external strain can effectively adjust the band gap and band edge positions of the GaAs monolayer.

### Carrier mobility

For an efficient catalytic material, high carrier mobility is necessary because it will benefit to suppressing the recombination of electron-hole pairs. The results of calculated carrier mobility and effective mass are summarized in Table 1. According to our calculated results, the GaAs monolayer has anisotropic effective mass. From Table 1, it is seen that the effective mass of electron (0.83*m*_o_) and hole (1.17 *m*_o_) along G-M direction are much larger than those of along G-K direction (0.29 *m*_o_ for electron, and 0.49 *m*_o_ for hole, where *m*_o_ is the mass of electron). Mobility along G-K direction (1053 and 2838 cm^2^V^−1^s^−1^ for electron and hole, respectively) are nearly three times larger than the value along the G-M direction (310 and 1043 cm^2^V^−1^s^−1^ for electron and hole), mainly because effective mass and deformation potential along the G-M direction are larger than the corresponding values along G-K direction. The highly anisotropic carrier mobility is favourable for long-term photocatalytic activity, because anisotropic carrier mobility can significantly reduce the recombination of photo-generated electron-hole pairs. The calculated carrier mobility value of GaAs monolayer which is much higher than the values of MoS_2_ monolayer (200 cm^2^V^−1^s^−1^)^42,43^, and photocatalysts monolayer PdPX (X = S, Se)^44^, is indicating that the carrier transfer in the GaAs monolayer is rather favourable.

**Table 1:** Calculated effective mass, stretching modulus, deformation potential, and carrier mobility.

Carrier type	m^*^/m_*o*_	*C*_*2D*_ (N/m)	*E*_*1*_ (eV)	*μ* (cm^2^V^−1^s^−1^)
Electron (G-M)	0.83	38.05	2.58	310
Electron (G-K)	0.29	38.05	2.37	1053
Hole (G-M)	1.17	38.05	0.95	1043
Hole (G-K)	0.49	38.05	0.89	2838

### OER and HER activity

**Figure 4:**
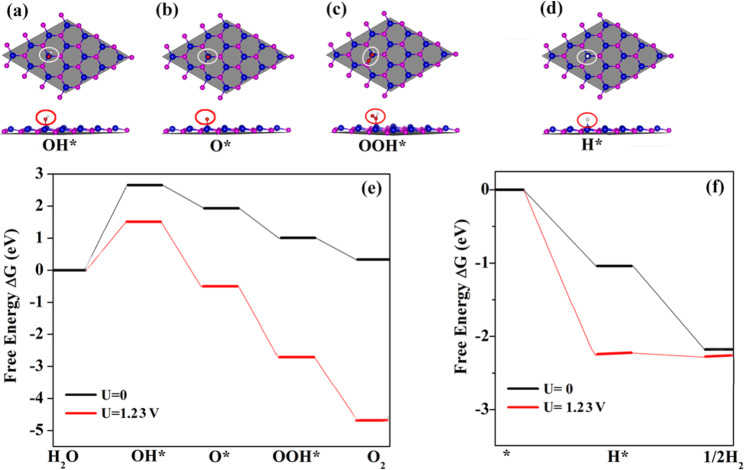
Photocatalytic pathway of the GaAs monolayer. (**a**–**d**) are absorbed intermediates, and (**e**,**f**) are reaction steps for OER and HER, respectively.

The band gap and band edge positions are not sufficient for predicting efficient photocatalytic water splitting properties. Therefore, to investigate further the water splitting activity, we examined the reaction free energy for water splitting in both dark and light radiated condition at neutral environment. At the present work, we calculated reaction free energy (Δ*G*) for both oxidation half reaction (OER) and hydrogen reduction half reaction (HER), and the corresponding results are summarized in Fig. 4. As is shown, the OER process includes four reaction steps with three absorbed intermediates (OH*, O*, OOH*), and the OER process includes two reaction steps with one absorbed intermediates (H*).

As for the OER (Fig. 4(e)), at the dark condition (*U* = 0), firstly, water molecule oxidized to the OH* by absorbing 2.65 eV energy. Secondly, OH* transferred to an O* by releasing 0.72 eV energy with electron and proton. Thirdly, O* by reacting with another H_2_O molecule forming OOH* with a Δ*G* of 0.92 eV. Finally, OOH* oxidized to O_2_ molecule by releasing one electron-photon pair with 0.68 eV energy. At the light radiation condition (*U* = 1.23 V), Δ*G* for all above four steps are downshifting, and the limiting potential is changed from 2.65 V to 1.39 V which is much lower than the limiting potential 2.28 V of g-C_3_N_4_^45^. When it comes to HER (Fig. 4(f)), under at both dark and light irradiated conditions, Δ*G* for all reaction steps are downshifting in the free-energy profile and belong to the exothermic process. The obtained negative free-energy profile demonstrates that GaAs monolayer will facilitate HER quite readily. The above results predicts that the GaAs monolayer has efficient water splitting properties.

### Optical properties

**Figure 5:**
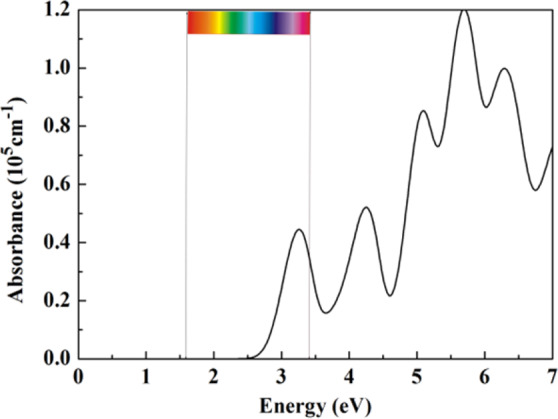
Optical absorption spectrum of GaAs monolayer.

Light harvesting performance of materials is another important requirement for photocatalytic activity. Therefore, to give an intuitive demonstration of light harvesting performance of the GaAs monolayer, we computed the absorbance using PBE0 hybrid function. Figure 5 shows the optical absorption coefficient of GaAs monolayer as a function of energy. As shown in the Fig. 5, prominent peaks appear in the energy region from 3 eV to 6 eV. It demonstrates that GaAs monolayer can absorbs the visible light and high energetic UV light. Meanwhile, we can see that the absorption of GaAs monolayer is rather strong (~10^5^ cm^−1^).

## Conclusions

In summary, in this work, density functional calculations are carried out for predicting photocatalytic water splitting activity of GaAs monolayer. We have accessed high kinetic stability based on phonon dispersion, cohesive energy and elastic constants. According to our computational results, the GaAs monolayer has a suitable width of band gap (2.56 eV), band edge position which can fulfil the water splitting requirements. Moreover, the GaAs monolayer has rather strong optical absorbance (~10^5^ cm^−1^) in the visible light and ultraviolet region. The calculated reaction free energy demonstrates its superiority in the readily occurrence of HER. More interestingly, the GaAs monolayer has a large anisotropic carrier mobility (2838 cm^2^V^−1^s^−1^), which can significantly reduce the recombination of photo-generated electrons and holes. These obtained electronic and optical properties indicate that the GaAs monolayer is a very promising photocatalytic water splitting energy transferring material that can convert solar energy to chemical energy. Additionally, compare to other monolayer photocatalysts, such as h-BN^11^, WS_2_^13^, and BP^15^, the GaAs monolayer possesses advantages on carrier mobility and visible light harvesting optical absorbance.

## Supplementary information

First Principles Calculation for Photocatalytic Activity of GaAs Monolayer.
