# Supplementary material for: First Principles Calculation for Photocatalytic Activity of GaAs Monolayer
Source: Sci Rep. 2020 Jun 12;10:9597. doi: 10.1038/s41598-020-66575-9 (PMC7293266; doi:10.1038/s41598-020-66575-9)
Supplement: Supplementary file 1 — First Principles Calculation for Photocatalytic Activity of GaAs Monolayer. [file 41598_2020_66575_MOESM1_ESM.docx]

**First Principles Calculation for Photocatalytic Activity of GaAs Monolayer**

Yilimiranmu Rouzhahong^#^, Mariyemu Wushuer^#^, Mamatrishat Mamat*, Qing Wang and Qian Wang

*School of Physics and technology, Xinjiang University, 666 Victory Road, Urumqi 830046, P. R. China*

**Corresponding Autho**r

*E-mail address: mmrishat@163.com

**Contents**

**S1. Phonon dispersion**

**S2. Impact of uniaxial stress to the CBM and VBM**

**S1. Phonon dispersion**





**Figure S1** The phonon spectra of the GaAs monolayer, there are no imaginary frequencies modes, which proves the good kinetic stability of the GaAs monolayer.

**S2. Impact of uniaxial stress to the CBM and VBM**







**Figure S2** Impacts of uniaxial strain along a and b direction to the CBM and VBM.
